# Association between functional Status and cardiac function in chronic heart failure: insights from the C-MIC II Trial

**DOI:** 10.1093/eschf/xvag102

**Published:** 2026-04-06

**Authors:** Marat Fudim, Tamara Kovacevic-Preradovic, Marija Zdravkovic, Sasko Jovev, Nermir Granov, Tanja Popov, Igor Rudez, Petar Vukovic, Velibor Ristic, Annette Holtdirk, Muhammad Shahzeb Khan, Faouzi Kallel, Miodrag Peric, Javed Butler, Stefan D Anker, Dragana Kosevic, J Eduardo Rame

**Affiliations:** Department of Medicine, Duke University, 2301 Erwin Road, Durham, NC 27710, USA; Duke Clinical Research Institute, Durham, NC, USA; Faculty of Medicine, University of Banja Luka, Banja Luka, Bosnia and Herzegovina; University Hospital Medical Center Bezanijska Kosa, Belgrade, Serbia; University Clinic of Cardiology, Skopje, North Macedonia; Clinical Center University of Sarajevo, Sarajevo, Bosnia and Herzegovina; Institute of Cardiovascular Diseases of Vojvodina, Vojvodina, Serbia; University Hospital Dubrava, Zagreb, Croatia; University Belgrade Medical School, Belgrade, Serbia; Cardiovascular Institute Dedinje, Belgrade, Serbia; RQM+, Frankfurt, Germany; Baylor Scott and White Health- Heart Hospital Plano, and Baylor Scott and White Research Institute, Dallas, TX, USA; Berlin Heals, Berlin, Germany; Cardiovascular Institute Dedinje, Belgrade, Serbia; University of Mississippi Medical Center, Jackson, MS, USA; Department of Cardiology (CVK) of German Heart Center Charité, German Centre for Cardiovascular Research (DZHK) partner site Berlin, Charité Universitätsmedizin, Berlin, Germany; Cardiovascular Institute Dedinje, Belgrade, Serbia; Bruce and Robbi Toll Heart and Vascular Institute, Thomas Jefferson University, Philadelphia, PA, USA

**Keywords:** Heart failure, Microcurrent therapy, LVEF, Functional status, Health status

## Abstract

**Introduction:**

Relationship between changes in cardiac function, functional capacity, and patient-reported health status in heart failure (HF) remains incompletely defined, which may help inform endpoint selection and clarify how distinct clinical domains reflect treatment response.

**Methods:**

This *post hoc* analysis of the randomized cardiac microcurrent (C-MIC) II trial, which evaluated the efficacy and safety of C-MIC therapy in patients with chronic HF with reduced ejection fraction on optimal guideline-directed medical therapy, included 65 ambulatory patients with non-ischaemic dilated cardiomyopathy, New York Heart Association (NYHA) Class III-IV symptoms, and baseline left ventricular ejection fraction (LVEF) 25–35%. Correlations between changes in Kansas City Cardiomyopathy Questionnaire Overall Summary Score (KCCQ-OSS), 6-minute walk distance (6MWD), core lab-assessed LVEF (primary measure) and site-assessed LVEF, and peak oxygen uptake (peak VO_2_) were evaluated at 4 weeks, 2 months, 3 months, 4 months, and 6 months using Pearson coefficients with 95% confidence intervals (CI).

**Results:**

The mean age was 60.0 ± 9.7 years and baseline LVEF was 29.8 ± 3.3%. Baseline 6MWD was 291.4 ± 61.6 m and KCCQ-OSS was 42.6 ± 22.7. From baseline to 6 months, changes in KCCQ-OSS (*n* = 63) and 6MWD (*n* = 61) showed modest correlations with core lab-assessed LVEF (*r* = 0.39; 95% CI: 0.16–0.58; *P* = .0015 and *r* = 0.39; 95% CI: 0.15–0.58; *P* = .0022, respectively). Changes in KCCQ-OSS and 6MWD correlated strongly (*n* = 62; *r* = 0.63; 95% CI: 0.46–0.76; *P* < .0001). Changes in KCCQ-OSS and 6MWD did not correlate significantly with changes in peak VO_2_ (*P* = .06 and *P* = .30, respectively). Changes in LVEF and peak VO_2_ (*n* = 55) demonstrated modest correlation (*r* = 0.41; 95% CI: 0.16–0.61; *P* = .002). Baseline correlations with peak VO_2_ were weak to modest but increased at 6 months for LVEF (*n* = 59; *r* = 0.56; 95% CI: 0.35–0.71; *P* < .0001).

**Conclusion:**

In advanced HF, improvements in health status and submaximal functional capacity associate modestly with LVEF, while LVEF correlates more closely with peak VO_2_. Cardiac function, functional capacity, and health status represent related but distinct domains, supporting multidimensional assessment in HF trials.

## Graphical Abstract

**Graphical Abstract:**
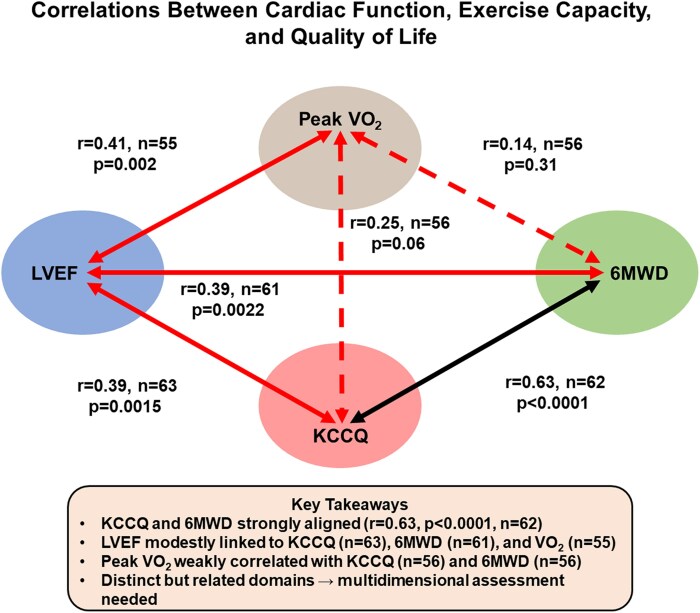
Solid black arrows indicate strong associations; solid red arrows indicate moderate associations; dashed red arrows indicate weak or non-significant associations.

## Introduction

Cardiac microcurrent (C-MIC) therapy is a novel, non-excitatory bioelectrical treatment designed to modulate myocardial electrophysiology and metabolism through the delivery of low-intensity direct electrical current. Preclinical studies have demonstrated its potential to enhance myocardial energy efficiency, reduce inflammation, and promote reverse remodelling,^1–3^ The C-MIC II trial evaluated the efficacy and safety of this therapy in patients with chronic heart failure (HF) with reduced ejection fraction (HFrEF) on optimal guideline-directed medical therapy (GDMT). The C-MIC II trial demonstrated that C-MIC therapy significantly improved left ventricular ejection fraction (LVEF), health status—as measured by the Kansas City Cardiomyopathy Questionnaire Overall Summary Score (KCCQ-OSS)—and functional capacity, as assessed by the six-minute walk distance (6MWD).^4^ Results from the two-year follow-up of the C-MIC pilot study further showed sustained improvements in cardiac function and functional capacity, reinforcing the long-term potential of this novel therapy.^5^ Collectively, these findings suggest that C-MIC may represent a promising new approach for improving outcomes in patients with limited remaining treatment options.

While significant improvements were observed across all pre-defined endpoints, the extent to which individual-level changes in cardiac function are reflected in functional and health status outcomes remains to be clarified. This is particularly important for patient-reported outcomes such as quality of life (QoL), which are known to be susceptible to placebo effects. Assessing their correlation with more objective parameters—such as LVEF, 6MWD, and cardiopulmonary exercise testing (CPET)— may help contextualize how these effects are reflected across different outcome domains. Understanding these inter-relationships is critical, as it may provide insight into the mechanisms by which C-MIC exerts its therapeutic benefit and clarify how different outcome measures reflect treatment response in both clinical practice and future research.

To better understand the relationship between improvements in cardiac function, functional capacity, and health status, we examined the relationships among three key outcome domains: LVEF, KCCQ-OSS and 6MWD. We also explored how these measures relate to peak oxygen consumption (peak VO_2_) to assess the extent to which gains in cardiopulmonary fitness align with improvements in cardiac function, exercise tolerance, and quality of life. These analyses were designed to provide a broader perspective on the multidimensional effect of C-MIC therapy and the consistency of responses across complementary clinical endpoints.

## Methods

### Patient population and follow-up visits

The patients included in this analysis represent the same cohort of 65 patients enrolled in the C-MIC II trial.^4^ The design of the C-MIC II trial has been described previously.^4^ Briefly, C-MIC II was a prospective, multicentre, open-label, randomized controlled trial designed to evaluate the safety and efficacy of cardiac microcurrent (C-MIC) therapy in patients with chronic HFrEF. The trial was sponsored by Berlin Heals GmbH and conducted in accordance with the principles of the Declaration of Helsinki. The study received all necessary regulatory and ethics committee approvals prior to initiation. All patients provided written informed consent before participation. The trial was prospectively registered on ClinicalTrials.gov (NCT04662034).

Study visits were conducted at baseline, 4 weeks, 2 months, 3 months, 4 months, and 6 months after randomization for both treatment and control groups. Echocardiographic assessments were performed at baseline, 4 weeks, 4 months, and 6 months, with images analysed by a blinded core laboratory to evaluate changes in LVEF. Cardiopulmonary exercise testing (CPET) was conducted at baseline, 4 months, and 6 months using the Bruce, modified Bruce, Naughton, modified Naughton, or RAMP protocol, according to site practice.

### Outcome measures

The primary measures used in the present analysis were LVEF assessed by the blinded core laboratory (primary measure) and by site investigators, functional capacity measured by the 6-minute walk distance (6MWD), patient-reported health status assessed by the Kansas City Cardiomyopathy Questionnaire Overall Summary Score (KCCQ-OSS) and Physical Limitation Score (KCCQ-PLS), and exercise capacity quantified by peak oxygen uptake (peak VO_2_) during CPET. The KCCQ-OSS is a validated, patient-reported 23-item instrument scored on a 0–100 scale that assesses symptoms (frequency and burden), physical limitations, social limitations, and quality of life in patients with HF; higher scores indicate better health status.^6^ Minimal clinically important differences (MCIDs) were considered to contextualize clinical relevance, including ∼5 points for small but meaningful change in KCCQ (with ≥10–20 points representing moderate to large improvement) and ∼20–30 m for 6MWD in HF populations.

### Statistical analysis

To assess the relationships among these domains, Pearson correlation coefficients (*r*) with 95% confidence intervals were calculated for absolute changes from baseline at each follow-up timepoint (4 weeks, 2, 3, 4, and 6 months). The correlation of 6-month changes was designated as the primary analysis. All other correlations across earlier timepoints were considered exploratory. Absolute change from baseline was selected because it is more directly interpretable from a clinical perspective and aligns with commonly used definitions of clinically meaningful change for endpoints such as LVEF, functional capacity, and patient-reported outcomes (e.g. KCCQ). Relative change measures can be unstable when baseline values are small and are less commonly used in this context. To account for potential influence of baseline values and regression to the mean, additional regression analyses for baseline values as covariates and sensitivity analyses using relative (percent) change from baseline were planned to assess the robustness of findings. Analyses were performed between KCCQ-OSS and core lab-assessed LVEF, and between 6MWD and core lab-assessed LVEF. Additional analyses between KCCQ-OSS and 6MWD, between KCCQ-OSS and site-assessed LVEF, and between 6MWD and site-assessed LVEF were also conducted. Correlations between both absolute values and changes in peak VO_2_ and the corresponding values for KCCQ-OSS, 6MWD, and LVEF at baseline, 4 months and 6 months were also examined. Scatter plots were generated to visualize these relationships and to identify potential trends or outliers. All statistical analyses were performed using SAS software, version 9.4 (SAS Institute, Cary, NC), and statistical significance was defined as *P* < .05. These analyses were exploratory in nature, and no formal adjustment for baseline characteristics or correction for multiple testing was applied to the correlation coefficients.

## Results

### Patient population

A total of 65 patients were randomized in a 1:1 ratio to receive either C-MIC therapy plus guideline-directed medical therapy (GDMT; *n* = 32) or GDMT alone (*n* = 33). The study cohort had a mean age 60.0 ± 9.7 years (range: 52–67), mean LVEF 29.81 ± 3.30% (range: 24.6–37.5) and was predominantly male (*Table 1*). All patients were receiving optimized guideline-directed medical therapy prior to randomization.

**Table 1:** Baseline characteristics^a^

Characteristics	*N* = 65	Range
Age, years	60.0 ± 9.7	52–67
Female sex (*N*, %)	19 (29.2)	
Body mass index (kg/m^2^)^b^	29.14 ± 3.93	20.8–35.9
Body surface area (m^2^)	2.06 ± 0.20	1.5–2.4
Type II diabetes (*N*, %)	11 (16.9)	
Hypertension (*N*, %)	46 (70.8)	
Lung disease (*N*, %)	11 (16.9)	
Kidney disease (*N*, %)	3 (4.6)	
GI disease (*N*, %)	5 (7.7)	
NYHA Class III/IV (*N*, %)	65 (100.0)	
KCCQ Overall Score	42.6 ± 22.67	2.9–93.2
Duration since initial HF diagnosis (year)	2.7 ± 1.5	1–4
QRS duration (ms)	112.3 ± 23.50	80–192
AF/flutter (*N*,%)	23 (35.4%)	
AV block (*N*,%)	7 (10.8%)	
LVEF (%)	29.81 ± 3.30	24.6–37.5
LVEDD (mm)	63.12 ± 4.78	52.0–73.0
LVESD (mm)	51.37 ± 5.93	40.0–66.0
ICD (*N*, %)	4 (6.2%)	
eGFR (mL/min/1.72cm^2^)	78.09 ± 23.65	34.2–176.0
6MWD, m	291.4 ± 61.58	192.0–476.0
Performed CPET? (*N*, %)	61 (93.8%)	
Peak VO_2_ (ml/kg/min)	15.9 ± 4.4	8.4–27.9
NT-proBNP (pg/mL)	1200.4 ± 1241.01	72.8–6219.0
SGLT2 inhibitors (*N*, %)	26 (40%)	
Beta-blockers (*N*, %)	63 (96.9%)	
RAAS inhibition (*N*, %)	64 (98.5%)	
Aldosterone antagonists (*N*, %)	64 (98.5%)	
Diuretics (*N*, %)	65 (100%)	

GI, gastrointestinal; NYHA, New York Heart Association; KCCQ, Kansas City Cardiomyopathy Questionnaire; HF, heart failure; AF, atrial fibrillation; AV, atrio-ventricular; LVEF, left ventricular ejection fraction; LVEDD, left ventricular end-diastolic dimension; LVESD, left ventricular end-systolic dimension; ICD, intracardiac defibrillator; eGFR estimated glomerular filtration; 6MWD, 6 minute walk distance; CPET, cardiopulmonary exercise test; Peak VO_2_, Peak oxygen consumption during CPET; NT-proBNP, *N*-terminal pro-B-type natriuretic peptide; RAAS, renin–angiotensin–aldosterone system; SGLT2, sodium–glucose co-transporter 2.

^a^± values are mean ± SD. There were no significant between-group differences in the characteristics at baseline.

^b^The body-mass index is the weight in kilograms divided by the square of the height in meters.

### Correlations between health Status, functional capacity, and cardiac function

**Figure 1:**
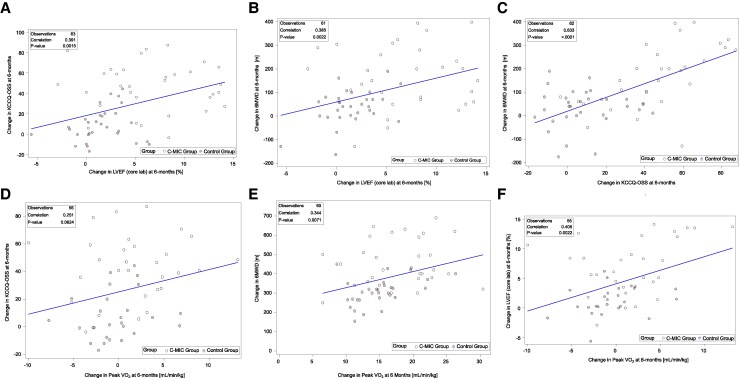
Associations between changes from baseline to 6 months in: (*A*) Kansas City Cardiomyopathy Questionnaire overall summary score (KCCQ-OSS) vs. left ventricular ejection fraction (LVEF; core lab-assessed), (*B*) six-minute walk distance (6MWD) vs. LVEF (core lab-assessed), (C) KCCQ-OSS vs. 6MWD, (D) KCCQ-OSS vs. peak oxygen consumption (peak VO_2_), (E) 6MWD vs. peak VO_2_, and (F) LVEF (core lab-assessed) vs. peak VO_2_

Across all follow-up visits, changes in KCCQ-OSS showed modest positive correlations with changes in LVEF whether assessed by the core lab (primary measure) or by the site investigators (*Table 2* and Supplementary Table S1 and S2). From baseline to 6 months, the correlation between changes in KCCQ-OSS and core lab–assessed LVEF was *r* = 0.39 (95% CI: 0.16–0.58; *P* = .0015) (*Figure 1A*, Graphical Abstract). When analysed separately by treatment group, correlations were directionally consistent, although coefficients were smaller and not statistically significant within individual groups. Similarly changes in 6MWD from baseline to 6 months demonstrated a modest positive correlation with the change in core lab–assessed LVEF (*r* = 0.39; 95% CI: 0.15–0.58; *P* = .0022) (*Figure 1B*, *Graphical Abstract*). In contrast, changes in KCCQ-OSS and 6MWD over the same period were more strongly correlated (*r* = 0.63; 95% CI: 0.46–0.76; *P* < .0001) (*Figure 1C*, *Graphical Abstract*).

**Table 2:** Pearson correlation coefficients between changes in KCCQ, 6MWD, core lab-assessed LVEF, and peak VO_2_ from baseline to 6 month visit

Variables compared	Timepoint	*R*	95% CI	*P*-value
ΔKCCQ-OSS vs ΔLVEF (core lab)	6 Months (*n* = 63)	0.39	(0.16, 0.58)	.0015
Δ6MWD vs ΔLVEF (core lab)	6 Months (*n* = 61)	0.39	(0.15, 0.58)	.0022
ΔKCCQ-OSS vs Δ6MWD	6 Months (*n* = 62)	0.63	(0.46, 0.76)	<.0001
ΔKCCQ-OSS vs ΔPeak VO_2_	6 Months (*n* = 56)	0.25	(−0.01, 0.48)	.0624
Δ6MWD vs ΔPeak VO_2_	6 Months (*n* = 56)	0.14	(−0.13, 0.39)	.3065
ΔLVEF vs ΔPeak VO_2_	6 Months (*n* = 55)	0.41	(0.16, 0.61)	.0022

Core lab-assessed LVEF was the primary analysis.

Δ, change from baseline; KCCQ, Kansas City Cardiomyopathy Questionnaire Overall Summary Score; 6MWD, 6-minute walk distance; LVEF, left ventricular ejection fraction; Peak VO_2_, peak oxygen uptake; CI, confidence interval.

### Correlations involving peak VO_2_

Changes from baseline to 6 months in KCCQ-OSS showed a modest, non-significant correlation with peak VO_2_ (*r* = 0.25; 95% CI: −0.01 to 0.48; *P* = .06), which was weaker than the corresponding correlation with 6MWD (*r* = 0.14; 95% CI: −0.13 to 0.39; *P* = .30) (*Figs 1D–E*, *Graphical Abstract*). In contrast, changes in LVEF and peak VO_2_ showed a modest positive correlation over the same period (*r* = 0.41, 95% CI: 0.16–0.61; *P* = .002) (*Figure 1F*, Graphical Abstract). The direction of these associations was generally similar across follow-up visits (Supplementary Table S2). In sensitivity analyses using regression models adjusting for baseline values, treatment group remained significantly associated with improvements across all endpoints (Supplementary Table S3).

### Correlation of peak VO_2_ with cardiac function, functional capacity, and health Status at baseline and 6 months

**Figure 2:**
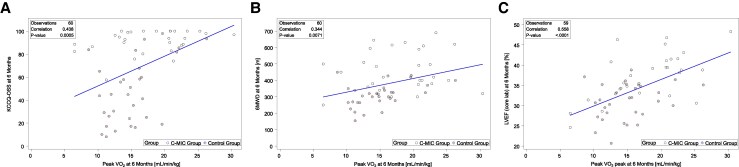
Associations at 6 months between: (*A*) Kansas City Cardiomyopathy Questionnaire overall summary score (KCCQ-OSS) and peak oxygen consumption (peak VO_2_), (*B*) six-minute walk distance (6MWD) and peak VO_2_, and (*C*) core lab-assessed left ventricular ejection fraction (LVEF) and peak VO_2_

At baseline, peak VO_2_ demonstrated weak-to-modest correlation with KCCQ-OSS (*r* = 0.27; 95% CI: 0.02–0.49; *P* = .033) and LVEF (*r* = .27; 95% CI: 0.02–0.51; *P* = .036), and was not significantly correlated with 6MWD (*r* = 0.16; 95% CI: −0.09 to 0.40; *P* = .20) (*Table 3* and Supplementary Table S4). By 6 months, these correlations increased in magnitude: KCCQ-OSS (*r* = 0.44; 95% CI: 0.21–0.62; *P* = .0005), 6MWD (*r* = 0.34; 95% CI: 0.10–0.55; *P* = .007), and LVEF (*r* = 0.56; 95% CI: 0.35–0.71; *P* < .0001) all showed stronger positive associations with peak VO_2_ (*Figs 2A–C*).

**Table 3:** Pearson correlation coefficients between KCCQ, 6MWD, LVEF, and peak VO_2_ at baseline and 6 month visit

Variables compared	Timepoint	r	95% CI	*P*-value
KCCQ-OSS vs Peak VO_2_	Baseline (*n* = 61)	0.27	(0.02, 0.49)	.0325
	6 Months (*n* = 60)	0.44	(0.21, 0.62)	.0005
6MWD vs Peak VO_2_	Baseline (*n* = 61)	0.16	(−0.09, 0.40)	.2042
	6 Months (*n* = 60)	0.34	(0.10, 0.55)	.0071
LVEF vs Peak VO_2_	Baseline (*n* = 61)	0.27	(0.02, 0.51)	.0327
	6 Months (*n* = 59)	0.56	(0.35, 0.71)	<.0001

KCCQ-OSS, Kansas City Cardiomyopathy Questionnaire Overall Summary Score; 6MWD, 6-minute walk distance; LVEF, left ventricular ejection fraction; Peak VO_2_, peak oxygen uptake; CI, confidence interval.

### Correlations between KCCQ-PLS and functional capacity

The correlation between KCCQ-PLS and measures of functional capacity (6MWD and peak VO_2_) was similar in magnitude to those observed for KCCQ-OSS, with comparable Pearson correlation coefficients and 95% CIs across timepoints (*Table 4* and *Table 5*, Supplementary Tables S5 and S6).

**Table 4:** Pearson correlation coefficients between KCCQ-OSS and peak VO_2_ and KCCQ-PLS and peak VO_2_ at baseline and 6 month visit

Variables compared	Timepoint	r	95% CI	*P*-value
KCCQ-OSS vs Peak VO_2_	Baseline (*n* = 61)	0.27	(0.02, 0.49)	.0325
	6 Months (*n* = 60)	0.44	(0.21, 0.62)	.0005
KCCQ-PLS vs Peak VO_2_	Baseline (*n* = 61)	0.27	(0.02, 0.49)	.0378
	6 Months (*n* = 60)	0.39	(0.15, 0.59)	.0019

KCCQ-OSS, Kansas City Cardiomyopathy Questionnaire Overall Summary Score; KCCQ-PLS, Kansas City Cardiomyopathy Questionnaire Physical Limitation Score; Peak VO_2_, peak oxygen uptake; CI, confidence interval.

**Table 5:** Pearson correlation coefficients between changes in KCCQ-OSS, and 6MWD and peak VO_2_ and KCCQ-PLS and 6MWD and peak VO_2_ from baseline to 6 month visit

Variables compared	Timepoint	R	95% CI	*P*-value
ΔKCCQ-OSS vs Δ6MWD	6 Months (*n* = 62)	0.63	(0.46, 0.76)	<.0001
ΔKCCQ-PLS vs Δ6MWD	6 Months (*n* = 62)	0.57	(0.37, 0.71)	<.0001
ΔKCCQ-OSS vs ΔPeak VO_2_	6 Months (*n* = 56)	0.25	(−0.01, 0.48)	.0624
ΔKCCQ-PLS vs ΔPeak VO_2_	6 Months (*n* = 56)	0.21	(−0.06, 0.45)	.1225

Δ, change from baseline; KCCQ-OSS, Kansas City Cardiomyopathy Questionnaire Overall Summary Score; KCCQ-PLS, Kansas City Cardiomyopathy Questionnaire Physical Limitation Score; 6MWD, 6-minute walk distance; LVEF, left ventricular ejection fraction; Peak VO_2_, peak oxygen uptake; CI, confidence interval.

## Discussion

In the C-MIC II trial, modest correlations were observed between LVEF and both patient-reported health status (KCCQ) and submaximal functional performance (6MWD) across all study visits, regardless of whether LVEF was assessed by the core lab (primary measure) or by site investigators. The correlation between KCCQ and 6MWD was consistently stronger than its association with LVEF or peak VO_2_, underscoring the closer alignment between patient-perceived health status and submaximal functional performance. Changes in KCCQ and 6MWD from baseline to 6 months were not significantly associated with changes in peak VO_2_, suggesting that individual-level gains in subjective and submaximal measures may not directly reflect improvements in maximal cardiopulmonary performance. In contrast, changes in LVEF were modestly but significantly correlated with changes in peak VO_2_, indicating some alignment between improvements in cardiac functions and exercise capacity.

At baseline, KCCQ and peak VO_2_ demonstrated a weak to modest correlation, which increased to a modest level by 6 months. Similarly, 6MWD showed no significant correlation with peak VO_2_ at baseline but reached a modest correlation at 6 months. The weaker correlations at baseline may, in part, reflect time discrepancies between baseline CPET, 6MWD, and KCCQ assessments, as well as variability introduced by the use of different CPET protocols across sites and patients.

These findings emphasize the importance of multidomain assessment when evaluating therapeutic response in HF. While improvements in LVEF, 6MWD, and KCCQ each provide valuable insights, their modest correlations with peak VO_2_ suggest that no single measure fully captures the complexity of functional recovery. Peak VO_2_, as a direct measure of maximal cardiopulmonary capacity, may yield unique information not adequately reflected by submaximal or patient-reported outcomes, reinforcing the value of incorporating objective exercise testing in trials of novel therapies such as C-MIC. KCCQ may be influenced by placebo effects in device trials. However, placebo effects are more likely to introduce variability rather than consistent alignment with objective measures. The observed correlations with LVEF and 6MWD therefore suggest that patient-reported improvements may, at least in part, reflect underlying physiological changes. Thus, correlations between change scores do not establish surrogacy or interchangeability among endpoints. The observed associations should be interpreted as reflecting related but distinct domains rather than evidence that LVEF or any single measure can serve as a surrogate endpoint. While both KCCQ and 6MWD may be influenced by placebo effects, the concordant associations across multiple domains support the interpretation that these improvements reflect meaningful changes in functional status rather than solely expectancy effects.

Numerous studies have examined the relationships among health status, functional capacity, and cardiac function in HF, focusing on correlations between KCCQ, LVEF, 6MWD, and peak VO_2_. Overall, the correlation between KCCQ scores and LVEF has been weak and inconsistent, with most studies reporting little to no significant association after adjusting for functional status or comorbidities.^7–13^

The foundational validation of the KCCQ by Green and colleagues established the KCCQ as a rigorous, sensitive, and responsive instrument for assessing health status in HF, demonstrating strong correlations with established measures and marked responsiveness to clinical improvement in decompensated patients (*r* = 0.46–0.74; mean change = 15–40 points).^7^ Our findings extend this seminal work by examining how changes in KCCQ-OSS relate to objective measures of cardiac function, submaximal functional performance, and peak exercise capacity. Improvements in KCCQ aligned modestly with enhancements in LVEF and 6MWD, and—to a lesser extent—with peak VO_2_, supporting the continued use of KCCQ as a sensitive health related QOL endpoint while reinforcing the value of integrated, multidomain assessment in HF trials.

In C-MIC II, the correlation between changes in KCCQ-OSS and 6MWD from baseline was stronger than that observed in COAPT, where Jain et al. reported a moderate 30-day correlation (*r* = 0.41) in patients with HF and secondary MR.^8^ Notably, in C-MIC II this stronger association was already evident at 4 weeks post–C-MIC and persisted at subsequent follow-up visits. In COAPT, moderate and large improvements in KCCQ corresponded to ≈25 m and ≈50 m gains in 6MWD, respectively, whereas small KCCQ improvements were not associated with measurable changes in walking distance. The earlier and stronger coupling seen in C-MIC II may reflect differences in patient population, intervention, and assessment windows, and underscores that while patient-reported and functional measures are related, their relationship can vary by clinical context.

Flynn and colleagues, using data from the HF-ACTION trial, reported modest correlations (*r* ≈ 0.18–0.34) between changes in patient-reported health status (KCCQ) and functional capacity measures (peak VO_2_ and 6MWD), and quantified a 5-point improvement in KCCQ as corresponding to clinically meaningful gains in exercise performance (∼2.5 mL·kg^−1^·min^−1^ in peak VO_2_ and ∼112 m in 6MWD).^9^ In our cohort, the observed improvements far exceeded these established MCID values, supporting that the correlated changes across domains reflect not only statistical associations but also clinically relevant benefits. Importantly, our analysis extends this prior work by also evaluating correlations with changes in LVEF, showing that improvements in patient-reported outcomes were modestly aligned with improvements in cardiac function, and that several of these associations strengthened over time.

In the PARALLAX trial—a large contemporary HFpEF cohort—baseline assessments revealed a modest relationship between QOL and functional capacity, with KCCQ-CSS and 6MWD showing a modest correlation (*r* ≈ 0.31), and the broader SF-36 physical functioning score correlating more strongly with exercise capacity (*r* ≈ 0.41).^10^ Multivariable analyses indicated that female sex, higher BMI, coronary artery disease, lower LVEF, and elevated NT-proBNP were associated with both worse QOL and reduced functional performance. These findings parallel our observations in HFrEF, where patient-reported health, functional capacity, and cardiac function each provide overlapping but distinct perspectives on treatment response, influenced by common patient characteristics.

The broader relevance of multidomain assessment across HF phenotypes is further highlighted by recent data in cardiomyopathy populations. In non-obstructive hypertrophic cardiomyopathy, Coats et al. examined the relationship between 6MWD and peak VO_2_, underscoring the growing interest in understanding how submaximal and maximal performance measures align in different HF subtypes.^11^ Our results complement this by showing modest correlations between 6MWD, KCCQ, LVEF, and peak VO_2_ in non-ischaemic HFrEF, with several strengthening over time (Supplementary Table S4). Conversely, in obstructive HCM, Bjerregaard et al. reported that CPET-derived functional capacity was not related to patient-reported health status, contrasting with our findings and suggesting that disease-specific factors such as dynamic outflow obstruction may uncouple symptomatic burden from measured exercise performance.^12^

Consistent with prior HF-ACTION data, which demonstrated modest baseline associations between KCCQ and both peak VO_2_ (*r* = 0.21) and 6MWD (*r* = 0.27), our cohort likewise showed weak-to-modest relationships at baseline (*Table 3*), reinforcing that patient-reported health status and objective exercise capacity capture overlapping but distinct constructs.^13^ Notably, in C-MIC II these relationships strengthened over follow-up, suggesting that alignment between perceived health and functional capacity may emerge as therapeutic effects consolidate over time. Importantly, the strength of these associations was similar for KCCQ-OSS and KCCQ-PLS, indicating that both domains provide comparable insights into functional capacity as measured by 6MWD and peak VO_2_.^13^ Together, our results underscore the value of multidomain assessment and provide temporal context: baseline correlations are modest, but clinically relevant improvements in health status can accompany gains in functional performance as treatment responses evolve.

Taken together, these data support the concept that while patient-reported outcomes like the KCCQ capture an important dimension of therapeutic benefit, they do not substitute for objective functional or cardiac measures. Each domain provides complementary information, and combined assessment offers the most comprehensive view of therapeutic effects in HF trials.

### Limitations

This study has several limitations that should be acknowledged. First, the sample size was relatively small, which may limit the statistical power to detect weaker associations and reduces the precision of correlation estimates. Second, some variables were missing at certain timepoints, most notably peak VO_2_, as CPET was performed in only 61 of the 65 enrolled patients. This missingness could reduce the representativeness of the findings for those endpoints. Missing CPET data were primarily due to logistical constraints, inability to perform maximal exercise, or site-specific factors rather than treatment assignment. Third, CPET was performed according to local site practice, using a variety of protocols (Bruce, modified Bruce, Naughton, modified Naughton, or RAMP). The lack of a standardized CPET protocol across sites and patients may have introduced variability in peak VO_2_ measurements, potentially attenuating observed correlations with other endpoints. Fourth, as an open-label study, the potential for placebo effects and other biases cannot be excluded, particularly for subjective endpoints such as patient-reported outcomes and functional measures. Echocardiographic measurements obtained at individual sites may have been more vulnerable to measurement variability or observer bias compared with the blinded core laboratory assessments, which likely explains the stronger correlations observed for site-assessed LVEF. Additionally, while echocardiographic images were analysed by a blinded core laboratory, the C-MIC device may have been visible on imaging, potentially unblinding readers and introducing bias into the centralized assessments. Moreover, CPET data were not analysed by a centralized core laboratory, which could have introduced inter-site variability in data acquisition and interpretation. An additional limitation of our study is the specific characteristics of the enrolled population, which was restricted to patients with non-ischaemic cardiomyopathy, a HF duration of less than five years, and an age younger than 65 years. The correlations observed in this analysis may not be generalizable to a broader ambulatory HF population, particularly those with ischaemic heart disease or a more chronic burden of HF. Several additional sensitivity analyses were considered but not fully pursued due to the modest sample size and limited data availability within specific subgroups, which may introduce additional variability and instability in the estimates, potentially limiting the interpretability of the results. Analyses evaluating the relationship between KCCQ-PLS and LVEF and further stratified sensitivity analyses involving CPET were constrained by incomplete data and reduced statistical power. These factors may also limit the ability to fully assess the influence of extreme values and subgroup-specific associations, and should be considered when interpreting the findings. Additionally, no formal correction for multiple testing was applied despite the large number of correlations evaluated, which increases the risk of type I error and false-positive findings. Consequently, these results should be interpreted as hypothesis-generating. Furthermore, while the primary correlation analyses were not adjusted for baseline characteristics, regression models adjusting for baseline values yielded consistent results. Nevertheless, the potential for residual confounding from other unmeasured variables cannot be entirely excluded. In addition, correlations derived from pooled analyses may, in part, reflect between-group differences driven by treatment effects rather than true within-subject associations across domains, and should therefore be interpreted with caution. Finally, given the multiple comparisons performed, emphasis should be placed on effect sizes and confidence intervals rather than individual *P*-values.

## Conclusion

In this analysis of the C-MIC II trial, improvements in patient-reported health status and functional capacity showed modest correlations with changes in core lab–assessed LVEF, while LVEF demonstrated a stronger association with peak VO_2_. These findings indicate that cardiac function, functional capacity, and health status represent related but distinct domains in HF, and observed correlations should not be interpreted as evidence of surrogacy or interchangeability. Multidimensional assessment incorporating imaging, functional testing, and patient-reported outcomes may provide a more comprehensive evaluation of therapeutic response in HF trials.

## Supplementary Material

xvag102_Supplementary_Data

## Declarations

### Data Availability

No data were generated or analysed for this manuscript.

### Pre-registered Clinical Trial Number

NCT04662034.
