# Supplementary material for: Association between functional Status and cardiac function in chronic heart failure: insights from the C-MIC II Trial
Source: ESC Heart Fail. 2026 Apr 6;13(2):xvag102. doi: 10.1093/eschf/xvag102 (PMC13126662; doi:10.1093/eschf/xvag102)
Supplement: xvag102_Supplementary_Data [file xvag102_supplementary_data.docx]

**Online Supplemental Material**

**Table S1**: Pearson Correlation Coefficients Between Changes in KCCQ, 6MWD, and site-assessed LVEF From Baseline to Follow-up Visits

**Table S2**: Pearson Correlation Coefficients Between Changes in KCCQ, 6MWD, core lab-assessed LVEF, and Peak VO2 From Baseline to Follow-up Visits

**Table S3**: Regression Analysis

**Table S4**: Pearson Correlation Coefficients Between KCCQ, 6MWD, LVEF, and Peak VO2 at Baseline and Follow-up Visits

**Table S5**: Pearson Correlation Coefficients Between KCCQ-OSS and Peak VO₂ and KCCQ-PLS and Peak VO_2_ at Baseline and Follow-up Visits

**Table S6**: Pearson Correlation Coefficients Between Changes in KCCQ-OSS, and 6MWD and Peak VO2 and KCCQ-PLS and 6MWD and Peak VO2 From Baseline to Follow-up Visits

| **Table S1. Pearson Correlation Coefficients Between Changes in KCCQ, 6MWD, and site-assessed LVEF From Baseline to Follow-up Visits** | | | | |
| --- | --- | --- | --- | --- |
| **Variables Compared** | **Timepoint** | **r** | **95% CI** | **p-value** |
| ΔKCCQ-OSS vs ΔLVEF (site) | 4 Weeks (n=64) | 0.42 | (0.19, 0.60) | 0.0006 |
|  | 2 Months (n=65) | 0.38 | (0.14, 0.57) | 0.0021 |
|  | 3 Months (n=62) | 0.42 | (0.19, 0.60) | 0.0007 |
|  | 4 Months (n=64) | 0.45 | (0.23, 0.62) | 0.0002 |
|  | 6 Months (n=64) | 0.46 | (0.24, 0.63) | 0.0001 |
| Δ6MWD vs ΔLVEF (site) | 4 Weeks (n=64) | 0.42 | (0.19, 0.60) | 0.0006 |
|  | 2 Months (n=65) | 0.44 | (0.22, 0.62) | 0.0002 |
|  | 3 Months (n=62) | 0.40 | (0.17, 0.59) | 0.0011 |
|  | 4 Months (n=64) | 0.48 | (0.26, 0.65) | 0.0001 |
|  | 6 Months (n=62) | 0.46 | (0.23, 0.63) | 0.0002 |
| Abbreviations: Δ, change from baseline; KCCQ, Kansas City Cardiomyopathy Questionnaire Overall Summary Score; 6MWD, 6-minute walk distance; LVEF, left ventricular ejection fraction; CI, confidence interval. | | | | |

| **Table S2. Pearson Correlation Coefficients Between Changes in KCCQ, 6MWD, core lab-assessed LVEF, and Peak VO_2_ From Baseline to Follow-up Visits** | | | | |
| --- | --- | --- | --- | --- |
| **Variables Compared** | **Timepoint** | **r** | **95% CI** | **p-value** |
| ΔKCCQ-OSS vs ΔLVEF (core lab) | 4 Weeks (n=62) | 0.33 | (0.09, 0.54) | 0.008 |
|  | 4 Months (n=61) | 0.20 | (-0.05, 0.43) | 0.1220 |
|  | 6 Months (n=63) | 0.39 | (0.16, 0.58) | 0.0015 |
| Δ6MWD vs ΔLVEF (core lab) | 4 Weeks (n=62) | 0.32 | (0.07, 0.52) | 0.0125 |
|  | 4 Months (n=61) | 0.38 | (0.14, 0.58) | 0.0025 |
|  | 6 Months (n=61) | 0.39 | (0.15, 0.58) | 0.0022 |
| ΔKCCQ-OSS vs Δ6MWD | 4 Weeks (n=64) | 0.65 | (0.48, 0.77) | <0.0001 |
|  | 2 Months (n=65) | 0.68 | (0.53, 0.79) | <0.0001 |
|  | 3 Months (n=62) | 0.67 | (0.50, 0.79) | <0.0001 |
|  | 4 Months (n=64) | 0.61 | (0.43, 0.74) | <0.0001 |
|  | 6 Months (n=62) | 0.63 | (0.46, 0.76) | <0.0001 |
| ΔKCCQ-OSS vs ΔPeak VO_2_ | 4 Months (n=53) | 0.25 | (-0.02, 0.49) | 0.0740 |
|  | 6 Months (n=56) | 0.25 | (-0.01, 0.48) | 0.0624 |
| Δ6MWD vs ΔPeak VO_2_ | 4 Months (n=53) | 0.16 | (-0.11, 0.42) | 0.2429 |
|  | 6 Months (n=56) | 0.14 | (-0.13, 0.39) | 0.3065 |
| ΔLVEF vs ΔPeak VO_2_ | 4 Months (n=51) | 0.17 | (-0.11, 0.43) | 0.2200 |
|  | 6 Months (n=55) | 0.41 | (0.16, 0.61) | 0.0022 |
| Abbreviations: Δ, change from baseline; KCCQ, Kansas City Cardiomyopathy Questionnaire Overall Summary Score; 6MWD, 6-minute walk distance; LVEF, left ventricular ejection fraction; Peak VO_2_, peak oxygen uptake; CI, confidence interval.  Note: Core lab–assessed LVEF was the primary analysis. | | | | |

| **Table S3: Regression analysis** | | | | | | | | |
| --- | --- | --- | --- | --- | --- | --- | --- | --- |
|  | | | | | | | **95% Confidence Interval** | |
| **Dependent variable** | **Variable** | **Estimate** | **Std. Error** | **t Value** | **p-value** | **Standardized estimate** | **lower limit** | **upper limit** |
| LVEF (core lab), change from baseline [%] | Intercept | -1.38364 | 4.531 | -0.31 | 0.7612 | 0.000 | -10.448 | 7.68043 |
|  | Treatment group | 5.11947 | 0.988 | 5.18 | <.0001 | 0.554 | 3.14401 | 7.09493 |
|  | LVEF (core lab) [%] at baseline | 0.09718 | 0.151 | 0.64 | 0.5229 | 0.069 | -0.2053 | 0.39964 |
| LVEF (site), change from baseline [%] | Intercept | -3.77457 | 6.818 | -0.55 | 0.5819 | 0.000 | -17.409 | 9.85953 |
|  | Treatment group | 8.09590 | 1.514 | 5.35 | <.0001 | 0.565 | 5.06811 | 11.1237 |
|  | LVEF (site) [%] at baseline | 0.10188 | 0.227 | 0.45 | 0.6550 | 0.047 | -0.3518 | 0.55553 |
| KCCQ PLS, change from baseline | Intercept | 31.54774 | 5.276 | 5.98 | <.0001 | 0.000 | 20.9987 | 42.0968 |
|  | Treatment group | 39.92283 | 5.064 | 7.88 | <.0001 | 0.692 | 29.7976 | 50.0481 |
|  | KCCQ PLS at baseline | -0.57790 | 0.102 | -5.69 | <.0001 | -0.499 | -0.7811 | -0.3747 |
| KCCQ OSS, change from baseline | Intercept | 26.39686 | 4.881 | 5.41 | <.0001 | 0.000 | 16.6365 | 36.1572 |
|  | Treatment group | 41.12137 | 4.351 | 9.45 | <.0001 | 0.747 | 32.4202 | 49.8225 |
|  | KCCQ OSS at baseline | -0.47052 | 0.098 | -4.82 | <.0001 | -0.381 | -0.6658 | -0.2753 |
| 6-min-walk distance, change from baseline [m] | Intercept | 238.00002 | 61.9 | 3.85 | 0.0003 | 0.000 | 114.145 | 361.855 |
|  | Treatment group | 151.38843 | 26.14 | 5.79 | <.0001 | 0.594 | 99.0825 | 203.694 |
|  | 6-min-walk distance at baseline [m] | -0.74620 | 0.213 | -3.51 | 0.0009 | -0.359 | -1.172 | -0.3204 |
| peakVO2 (weight adjusted), change from baseline [mL/min/kg body weight] | Intercept | 4.05576 | 2.008 | 2.02 | 0.0485 | 0.000 | 0.02805 | 8.08347 |
|  | Treatment group | 2.28400 | 1.063 | 2.15 | 0.0362 | 0.272 | 0.15233 | 4.41566 |
|  | peakVO2 (weight adjusted) at baseline [mL/min/kg body weight] | -0.28651 | 0.119 | -2.41 | 0.0195 | -0.304 | -0.5251 | -0.048 |

| **Table S4. Pearson Correlation Coefficients Between KCCQ, 6MWD, LVEF, and Peak VO_2_ at Baseline and Follow-up Visits** | | | | |
| --- | --- | --- | --- | --- |
| **Variables Compared** | **Timepoint** | **r** | **95% CI** | **p-value** |
| KCCQ-OSS vs Peak VO_2_ | Baseline (n=61) | 0.27 | (0.02, 0.49) | 0.0325 |
|  | 4 Months (n=56) | 0.42 | (0.18, 0.62) | 0.0012 |
|  | 6 Months (n=60) | 0.44 | (0.21, 0.62) | 0.0005 |
| 6MWD vs Peak VO_2_ | Baseline (n=61) | 0.16 | (-0.09, 0.40) | 0.2042 |
|  | 4 Months (n=56) | 0.29 | (0.02, 0.51) | 0.0327 |
|  | 6 Months (n=60) | 0.34 | (0.10, 0.55) | 0.0071 |
| LVEF vs Peak VO_2_ | Baseline (n=61) | 0.27 | (0.02, 0.51) | 0.0327 |
|  | 4 Months (n=54) | 0.34 | (0.08, 0.56) | 0.0115 |
|  | 6 Months (n=59) | 0.56 | (0.35, 0.71) | <0.0001 |
| Abbreviations: KCCQ-OSS, Kansas City Cardiomyopathy Questionnaire Overall Summary Score; 6MWD, 6-minute walk distance; LVEF, left ventricular ejection fraction; Peak VO_2_, peak oxygen uptake; CI, confidence interval. | | | | |

| **Table S5. Pearson Correlation Coefficients Between KCCQ-OSS and Peak VO₂ and KCCQ-PLS and Peak VO_2_ at Baseline and Follow-up Visits** | | | | |
| --- | --- | --- | --- | --- |
| **Variables Compared** | **Timepoint** | **r** | **95% CI** | **p-value** |
| KCCQ-OSS vs Peak VO_2_ | Baseline (n=61) | 0.27 | (0.02, 0.49) | 0.0325 |
|  | 4 Months (n=56) | 0.42 | (0.18, 0.62) | 0.0012 |
|  | 6 Months (n=60) | 0.44 | (0.21, 0.62) | 0.0005 |
| KCCQ-PLS vs Peak VO_2_ | Baseline (n=61) | 0.27 | (0.02, 0.49) | 0.0378 |
|  | 4 Months (n=56) | 0.39 | (0.14, 0.59) | 0.0031 |
|  | 6 Months (n=60) | 0.39 | (0.15, 0.59) | 0.0019 |
| Abbreviations: KCCQ-OSS, Kansas City Cardiomyopathy Questionnaire Overall Summary Score; KCCQ-PLS, Kansas City Cardiomyopathy Questionnaire Physical Limitation Score; Peak VO_2_, peak oxygen uptake; CI, confidence interval. | | | | |

| **Table S6. Pearson Correlation Coefficients Between Changes in KCCQ-OSS, and 6MWD and Peak VO_2_ and KCCQ-PLS and 6MWD and Peak VO_2_ From Baseline to Follow-up Visits** | | | | |
| --- | --- | --- | --- | --- |
| **Variables Compared** | **Timepoint** | **r** | **95% CI** | **p-value** |
| ΔKCCQ-OSS vs Δ6MWD | 4 Weeks (n=64) | 0.65 | (0.48, 0.77) | <0.0001 |
|  | 2 Months (n=65) | 0.68 | (0.53, 0.79) | <0.0001 |
|  | 3 Months (n=62) | 0.67 | (0.50, 0.79) | <0.0001 |
|  | 4 Months (n=64) | 0.61 | (0.43, 0.74) | <0.0001 |
|  | 6 Months (n=62) | 0.63 | (0.46, 0.76) | <0.0001 |
| ΔKCCQ-PLS vs Δ6MWD | 4 Weeks (n=64) | 0.56 | (0.36, 0.71) | <0.0001 |
|  | 2 Months (n=65) | 0.58 | (0.39, 0.72) | <0.0001 |
|  | 3 Months (n=62) | 0.61 | (0.43, 0.75) | <0.0001 |
|  | 4 Months (n=64) | 0.56 | (0.36, 0.71) | <0.0001 |
|  | 6 Months (n=62) | 0.57 | (0.37, 0.71) | <0.0001 |
| ΔKCCQ-OSS vs ΔPeak VO_2_ | 4 Months (n=53) | 0.25 | (-0.02, 0.49) | 0.0740 |
|  | 6 Months (n=56) | 0.25 | (-0.01, 0.48) | 0.0624 |
| ΔKCCQ-PLS vs ΔPeak VO_2_ | 4 Months (n=53) | 0.21 | (-0.07, 0.45) | 0.1335 |
|  | 6 Months (n=56) | 0.21 | (-0.06, 0.45) | 0.1225 |
| Abbreviations: Δ, change from baseline; KCCQ-OSS, Kansas City Cardiomyopathy Questionnaire Overall Summary Score; KCCQ-PLS, Kansas City Cardiomyopathy Questionnaire Physical Limitation Score; 6MWD, 6-minute walk distance; LVEF, left ventricular ejection fraction; Peak VO_2_, peak oxygen uptake; CI, confidence interval. | | | | |
